# Changing spatiotemporal patterns for hepatitis of unspecified aetiology in China, 2004–2021: a population-based surveillance study

**DOI:** 10.3389/fpubh.2023.1177965

**Published:** 2023-05-05

**Authors:** Na Zhao, Xiangyu Guo, Lan Wang, Hongming Zhou, Lei Gong, Ziping Miao, Yijuan Chen, Shuwen Qin, Zhao Yu, Shelan Liu, Supen Wang

**Affiliations:** ^1^School of Ecology and Environment, Anhui Normal University, Wuhu, Anhui Province, China; ^2^Institute of Zoology, Chinese Academy of Sciences, Beijing, China; ^3^Department of Neuro-Oncology, Cancer Center, Beijing Tiantan Hospital, Capital Medical University, Beijing, China; ^4^Department of Geriatrics, The First Affiliated Hospital, Zhejiang University School of Medicine, Hangzhou, Zhejiang Province, China; ^5^Key Laboratory of Diagnosis and Treatment of Aging and Physic-Chemical Injury Diseases of Zhejiang Province, Hangzhou, Zhejiang Province, China; ^6^The School of Basic Medical Sciences, Wannan Medical College, Wuhu, Anhui Province, China; ^7^Anhui Provincial Center for Disease Control and Prevention, Hefei, Anhui Province, China; ^8^Department of Infectious Diseases, Zhejiang Provincial Center for Disease Control and Prevention, Hangzhou, Zhejiang Province, China; ^9^College of Life Sciences, Anhui Normal University, Wuhu, Anhui Province, China

**Keywords:** epidemiological features, hepatitis of unspecified aetiology, incidence, mortality, spatio-temporal pattern

## Abstract

**Objectives:**

As global efforts continue toward the target of eliminating viral hepatitis by 2030, the emergence of acute hepatitis of unspecified aetiology (HUA) remains a concern. This study assesses the overall trends and changes in spatiotemporal patterns in HUA in China from 2004 to 2021.

**Methods:**

We extracted the incidence and mortality rates of HUA from the Public Health Data Center, the official website of the National Health Commission of the People’s Republic of China, and the National Notifiable Infectious Disease Surveillance System from 2004 to 2021. We used R software, ArcGIS, Moran’s statistical analysis, and joinpoint regression to examine the spatiotemporal patterns and annual percentage change in incidence and mortality of the HUA across China.

**Results:**

From 2004 to 2021, a total of 707,559 cases of HUA have been diagnosed, including 636 deaths. The proportion of HUA in viral hepatitis gradually decreased from 7.55% in 2004 to 0.72% in 2021. The annual incidence of HUA decreased sharply from 6.6957 per 100,000 population in 2004 to 0.6302 per 100,000 population in 2021, with an average annual percentage change (APC) reduction of −13.1% (*p* < 0.001). The same result was seen in the mortality (APC, −22.14%, from 0.0089/100,000 in 2004 to 0.0002/100,000 in 2021, *p* < 0.001). All Chinese provinces saw a decline in incidence and mortality. Longitudinal analysis identified the age distribution in the incidence and mortality of HUA did not change and was highest in persons aged 15–59 years, accounting for 70% of all reported cases. During the COVID-19 pandemic, no significant increase was seen in pediatric HUA cases in China.

**Conclusion:**

China is experiencing an unprecedented decline in HUA, with the lowest incidence and mortality for 18 years. However, it is still important to sensitively monitor the overall trends of HUA and further improve HUA public health policy and practice in China.

## Introduction

Hepatitis is an inflammation of the liver caused by various infectious viruses and noninfectious agents (e.g., alcohol use, toxins, medications, and certain medical conditions) that can cause multiple health problems, some of which can be fatal (1–3). Viral hepatitis is the most common type of liver inflammation, with five main virus strains, referred to as types A, B, C, D, and E (4). Hepatitis of unspecified aetiology (HUA) is defined as viral hepatitis in which common viral hepatitis agents (HAV, HBV, HCV, HDV, and HEV) are ruled out by laboratory investigations. Each year, viral hepatitis affects millions of people worldwide and is a major public health threat and a leading cause of death globally. Highly effective preventive vaccines and treatments have made the global elimination of viral hepatitis by 2030, a realistic goal endorsed by all WHO member states in 2016 (5–7).

While scientists focused on efforts to reduce hepatitis-related mortality by 65% and new infections by 90% by 2030 globally, an unexpected acute HUA emerged in the young children, 2022, and quickly spread to many countries (8–10). The United Kingdom first reported an increase in acute HUA not caused by hepatitis types A, B, C, D, or E among previously healthy children aged under 10 years (11–14). As of November 24, 2022, 572 cases of acute HUA have been reported in 22 countries (15). The current leading hypotheses concern adeno-associated virus serotype 2 (AAV2) and adenovirus involvement, possibly with an immunological cofactor that is triggering more severe infection or immune-mediated liver damage (15). This unusual public health occurrence increased rapidly raised concerns about HUA internationally.

Since China has the heaviest burden of viral hepatitis in the world, to respond to the potential threat of HUA, it is necessary to establish baseline criteria in retrospective cohorts and define historical levels of HUA (16). Our aim was to describe a spatiotemporal epidemiology of the HUA over 18 years in China. We expect this study will contribute to a better understanding of the overall trend of HUA in China, increased vigilance for this potentially lethal disease beyond the current epidemic, and ultimately, early detection and control of this fatal disease, with improved clinical outcomes.

## Methods

### Nationally notifiable diseases in China

In response to the 2003 severe acute respiratory syndrome (SARS) outbreak, the Chinese government established a web-based reporting system for selected infectious diseases in real time, called the National Surveillance System for Infectious Diseases (NIDSS) (17). This system covers the Chinese population of 1.4 billion across 31 provinces in China. Nationally notifiable diseases include 40 infectious diseases, which are classified according to prevalence or great harm by decreasing severity into classes A, B, and C. Viral hepatitis, which belongs to Class B, is further divided into hepatitis A, hepatitis B, hepatitis C, hepatitis D, hepatitis E, and HUA.

### Case diagnosis criteria

National Surveillance System for Infectious Diseases data included clinically diagnosed cases, suspected cases, or confirmed cases of HUA, which including cases may be due to a viral hepatitis agent or agents not yet identified and are currently classified as non-ABCDE. The case definitions and diagnostic criteria were approved and issued by the China National Standardization Administration Committee.^1^

### Data source

The data on five types of viral hepatitis were obtained from the National Reporting System for Infectious Diseases and various publicly available sources, including the National Health Commission of the People’s Republic of China, the Public Health Science Data Center of the Chinese Center for Disease Control and Prevention,^2^ and local health authorities’ news releases, ProMed posts, and published literature. The annual population data for the years 2004 to 2021 were collected from the data published on the website of the Chinese National Bureau of Statistics^3^ at the end of each year (18).

### Data collection

We obtained HUA incidence and mortality data covering the period from 2004 to 2021, stratified by date (month and year), patient age, and province. To further assess the proportions of HUA in all reported cases of viral hepatitis, we also extracted the monthly and yearly cases and deaths of hepatitis A, hepatitis B, hepatitis C, and hepatitis E. Because of the lack of records on HDV infection prior to 2016 and small sample sizes, we excluded it from further analysis (19). We defined annual incidence (per 100,000) as the number of annual incident cases divided by the population size and annual mortality (per 100,000) as the number of deaths per year divided by the total population size.

### Statistical analysis

We used the R package (version 4.2.2), Joinpoint Regression Analysis software (version 4.5.0.1; National Cancer Institute), ArcGIS 10.2 (ArcMap, version 10.3; ESRI Inc., Redlands, CA, United States), and SaTScan.9.6^4^ to analyze the temporal trend, the annual percentage change (APC), and the spatiotemporal distribution of the incidence and mortality rate of the HUA. The joinpoint regression models were used to verify trends in HUA between 2004 and 2021 (20–23). Moran’s statistics were used for spatial autocorrelation domains between different provinces from 2004 to 2020. Queen contiguity weights were constructed for determining neighbors, and Monte Carlo randomization was used for testing the significance of Moran’s statistics, with the null hypothesis that the associated rates of HUA in mainland China are completely randomly distributed (24–26). Furthermore, we applied correlational analysis to ascertain whether correlations with other types of viral hepatitis were associated with HUA. Pearson product–moment coefficient and coefficient of determination (linear regression) were used to assess the similarity of these types of viral hepatitis to each other.

### Ethical approval

All data were obtained from publicly available data sources. All data were supplied and analyzed in an anonymous manner, without access to personal identifying information.

## Results

### Overall trends in yearly incidence and mortality of HUA, 2004–2021

**Figure 1:**
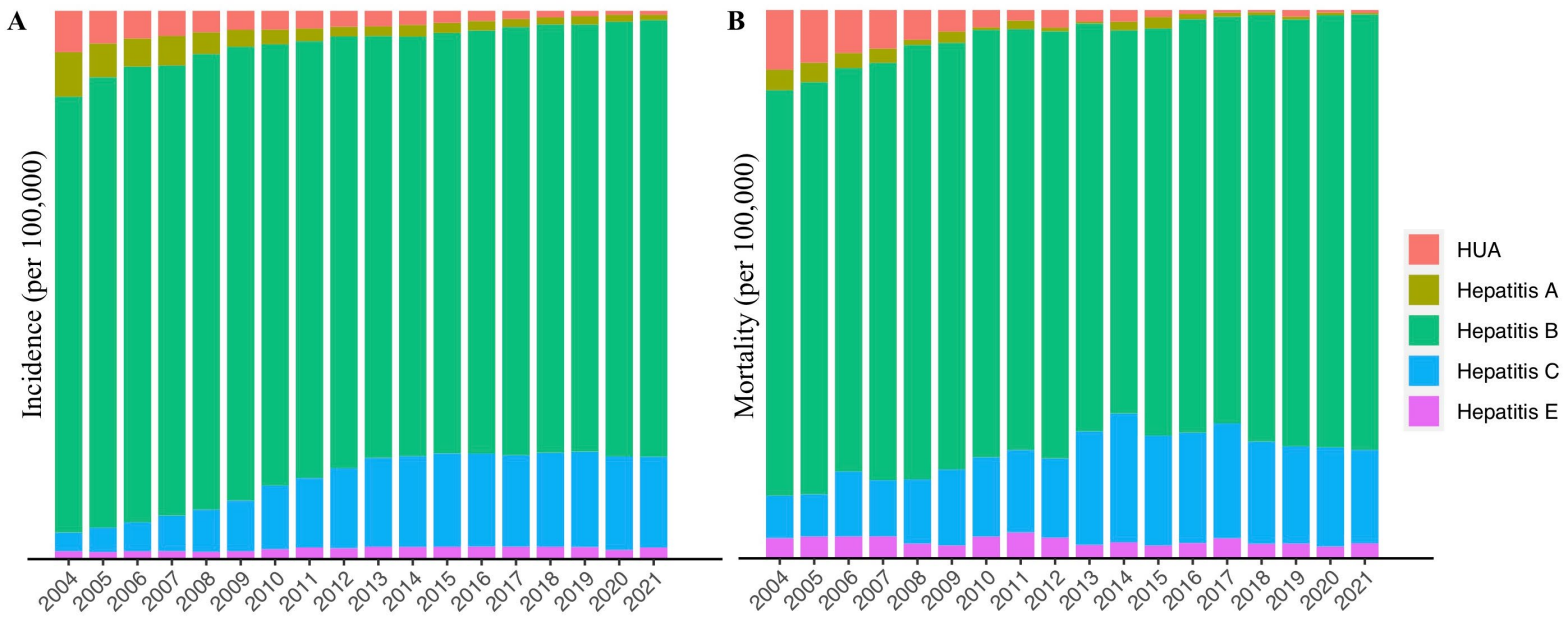
Proportion of hepatitis of unspecified aetiology in viral hepatitis: 2004–2021. **(A)** Incidence rate. **(B)** Mortality rate. HUA, hepatitis of unspecified aetiology.

**Figure 2:**
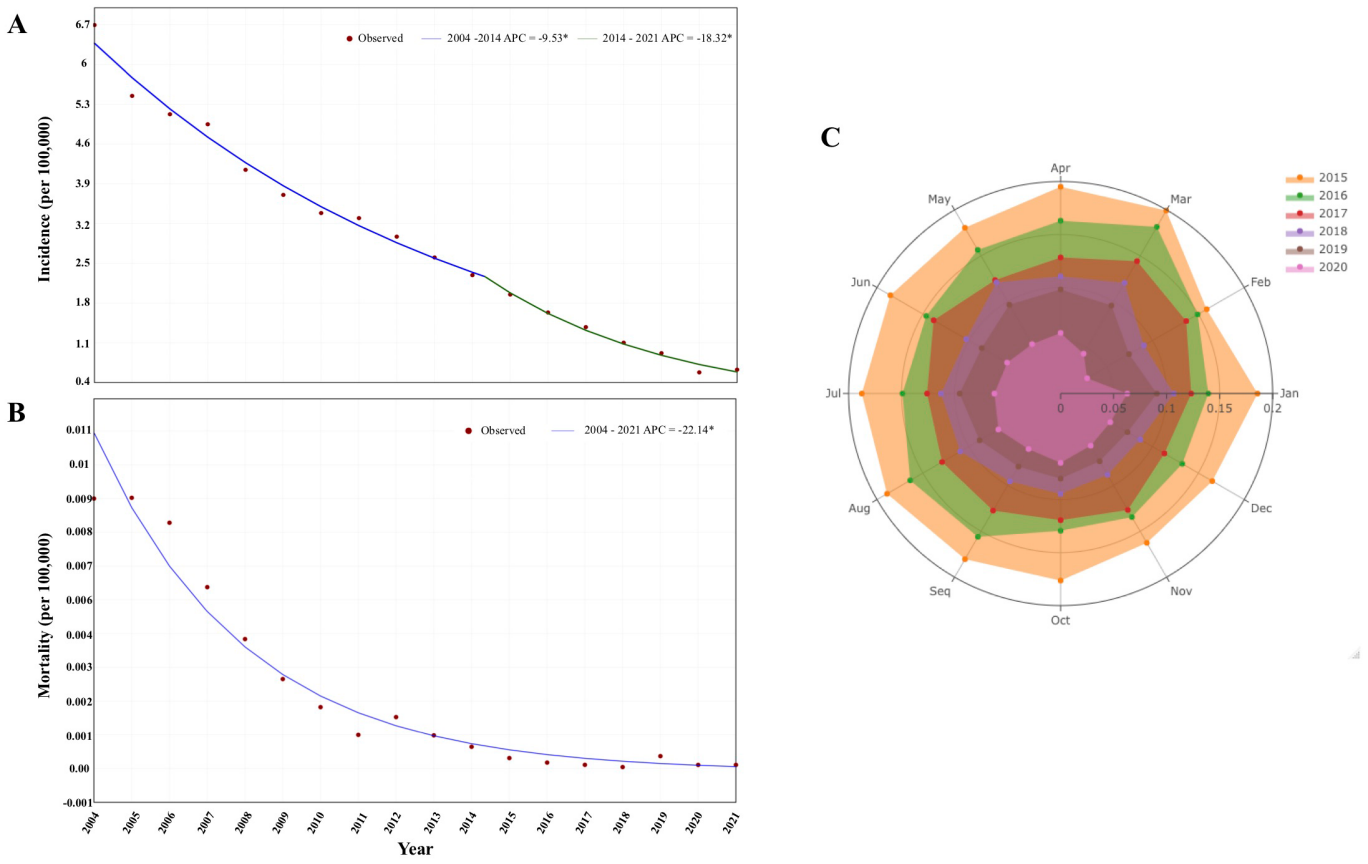
Temporal trends in the incidence and mortality from hepatitis of unspecified aetiology. **(A)** Joinpoint regression showing trends in the incidence of HUA, 2004–2021. **(B)** Joinpoint regression showing trends in the mortality of HUA, 2004–2021. **(C)** Seasonality trends of HUA, January 2016–December 2020. ^*^Statistically significant trends. APC, annual percentage change; HUA, hepatitis of unspecified aetiology.

A total of 707,559 HUA cases were diagnosed during this period, including 636 deaths. The proportion of HUA in viral hepatitis gradually decreased over the following 18 years, from 7.55% in 2004 to 0.72% in 2021 (Figure 1A). In accordance with that, the incidence rate of HUA decreased from 6.6957 (per 100,000 population) in 2004 to 0.6302 in 2021 in the mainland China. And the change of constituent ratio of etiological confirmed cases and the change of HUE incidence rate was significantly correlated (*R* = 0.8315, *p* < 0.001) in these years (Supplementary Figure 1). Furthermore, joinpoint regression indicated that the long-term trends in the APC of yearly incidence could be divided into two stages (Figure 2A). From 2004 to 2014, the yearly incidence of the HUA decreased significantly, from 6.6957 per 100,000 population in 2004 to 2.3288 per 100,000 population in 2014; the APC decreased by −9.53% [95% confidence interval (CI), −10.3 to −8.7%; *p* < 0.001]. From 2014 to 2021, the yearly incidence of HUA decreased from 2.3288 per 100,000 population in 2014 to 0.6302 per 100,000 population in 2021, and the APC was −18.32% (95% CI, −21.0% to −15.5%; *p* < 0.001). Additionally, we detected the seasonality trends of the HUA based on the available data of monthly incidence from January 2015 to December 2020. Although there was virtually no seasonality discovered during this stage, the incidence rate in February and March 2020 were relatively decreased (Figure 2C).

As for the mortality rate in these years, a similar decline of mortality in the proportion of viral hepatitis was also found during this period, from 10.86% in 2004 to 0.54% in 2021, although with slight fluctuations (Figure 1B). Joinpoint regression indicated that the yearly HUA mortality rate decreased steadily (no jointpoints were identified) from 0.0089 per 100,000 population in 2004 to 0.0002 per 100,000 population in 2021, with an APC reduction of −22.14% (95% CI, −24.5 to −19.7%; *p* < 0.001, Figure 2B).

### Geographical distribution of HUA, 2004–2020

**Figure 3:**
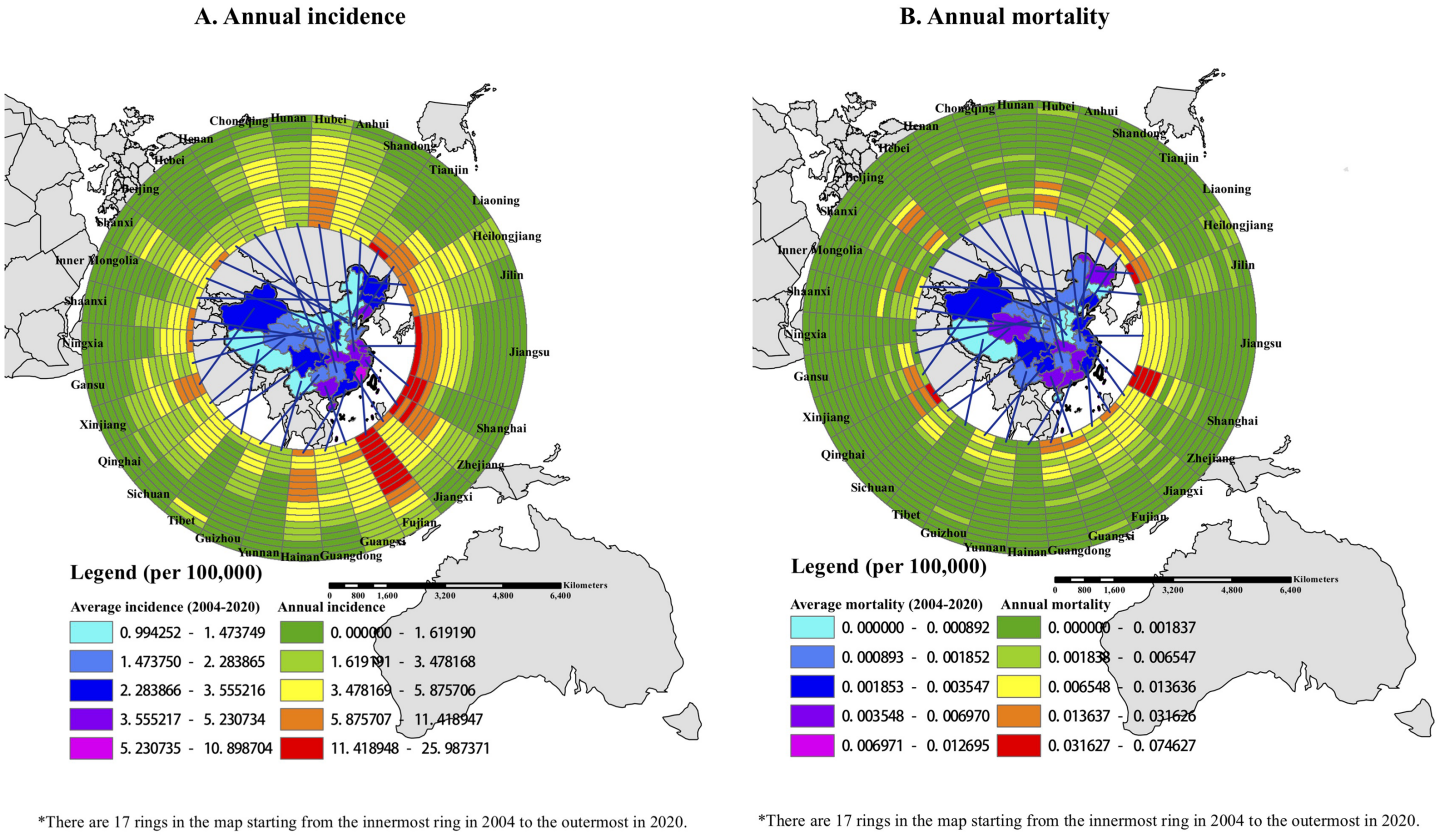
Spatiotemporal distribution of incidence and mortality due to hepatitis of unspecified aetiology in 31 province-level units: 2004–2020. **(A)** Spatiotemporal clusters for HUA incidence. **(B)** Spatiotemporal clusters for HUA mortality. All 17 years of incidence data for 31 provinces were used, with a maximum cluster population size of 10% to minimize false clusters, and a maximum temporal window of 3 years to examine the clusters. Local risk ring maps were created using ArcGIS software. The 17 rings contain data for each year studied, with the innermost ring bearing data for 2004 and moving outward through the years to the outermost ring bearing data for 2020.

From 2004 to 2020, all 31 provinces in mainland China reported HUA cases. Although the annual incidence and mortality in all the provinces generally showed downward trends (Table 1), the average yearly incidence was heterogeneously distributed across 31 provinces in mainland China. The average yearly incidence was highest in Fujian province, at 10.8987 per 100,000 people (Figure 3A). Shanghai, Qinghai, and Heilongjiang had relatively high annual mortality rates in the first few years, while Ningxia, Tibet, and Hainan provinces reported no fatal HUA cases during these years (Figure 3B). It was worth mentioning that Fujian province had both the highest annual incidence and the highest average annual incidence for most of these years.

**Table 1:** Changes in incidence and mortality (per 100,000) for HUA in China, 2004 vs. 2020.

Region	Annual incidence (per 100,000)					Annual mortality (per 100,000)				
	2004		2020		Overall trends (2020 vs. 2004)	2004		2020		Overall trends (2020 vs. 2004)
	Cases	Incidence	Cases	Incidence		Deaths	Mortality	Deaths	Mortality	
Total	87,036	6.6957	8,203	0.5809	−91.32%	115	0.0088	3	0.0002	−97.60%
Beijing	1,389	9.3034	13	0.0594	−99.36%	0	0.0000	0	0.0000	NA
Tianjin	1,265	12.3535	0	0.0000	−100.00%	2	0.0195	0	0.0000	−100.00%
Hebei	3,076	4.5176	135	0.1809	−96.00%	1	0.0015	0	0.0000	−100.00%
Shanxi	1,345	4.0330	248	0.7106	−82.38%	0	0.0000	0	0.0000	NA
Inner Mongolia	954	4.0017	75	0.3121	−92.20%	2	0.0084	0	0.0000	−100.00%
Liaoning	3,739	8.8665	700	1.6451	−81.45%	5	0.0119	1	0.0024	−80.18%
Jilin	1,746	6.4452	18	0.0750	−98.84%	0	0.0000	0	0.0000	NA
Heilongjiang	2,845	7.4535	148	0.4667	−93.74%	15	0.0393	0	0.0000	−100.00%
Shanghai	4,527	25.9874	12	0.0482	−99.81%	13	0.0746	0	0.0000	−100.00%
Jiangsu	9,117	12.2656	624	0.7361	−94.00%	6	0.0081	0	0.0000	−100.00%
Zhejiang	7,396	15.6695	283	0.4375	−97.21%	6	0.0127	0	0.0000	−100.00%
Anhui	3,600	5.5719	1,239	2.0295	−63.58%	2	0.0031	1	0.0016	−47.08%
Fujian	7,495	21.3472	695	1.6703	−92.18%	4	0.0114	0	0.0000	−100.00%
Jiangxi	3,269	7.6307	238	0.5267	−93.10%	6	0.0140	0	0.0000	−100.00%
Shandong	3,845	4.1885	440	0.4329	−89.67%	3	0.0033	0	0.0000	−100.00%
Henan	3,125	3.2160	42	0.0422	−98.69%	3	0.0031	0	0.0000	−100.00%
Hubei	4,381	7.2822	412	0.7171	−90.15%	7	0.0116	0	0.0000	−100.00%
Hunan	1,443	2.1544	543	0.8172	−62.07%	2	0.0030	0	0.0000	−100.00%
Guangdong	4,204	5.0626	781	0.6187	−87.78%	15	0.0181	1	0.0008	−95.61%
Guangxi	2,591	5.2997	831	1.6557	−68.76%	5	0.0102	0	0.0000	−100.00%
Hainan	507	6.1980	93	0.9190	−85.17%	0	0.0000	0	0.0000	NA
Chongqing	1,689	5.4100	70	0.2181	−95.97%	2	0.0064	0	0.0000	−100.00%
Sichuan	4,833	5.5393	288	0.3440	−93.79%	5	0.0057	0	0.0000	−100.00%
Guizhou	1,601	4.1009	69	0.1788	−95.64%	4	0.0102	0	0.0000	−100.00%
Yunnan	1,213	2.7475	14	0.0296	−98.92%	0	0.0000	0	0.0000	NA
Tibet	148	5.4015	2	0.0546	−98.99%	0	0.0000	0	0.0000	NA
Shaanxi	2,236	6.0351	77	0.1947	−96.77%	4	0.0108	0	0.0000	−100.00%
Gansu	1,064	4.0626	41	0.1639	−95.96%	0	0.0000	0	0.0000	NA
Qinghai	230	4.2672	18	0.3035	−92.89%	2	0.0371	0	0.0000	−100.00%
Ningxia	397	6.7517	10	0.1387	−97.95%	0	0.0000	0	0.0000	NA
Xinjiang	1,766	8.9964	44	0.1699	−98.11%	1	0.0051	0	0.0000	−100.00%

**Figure 4:**
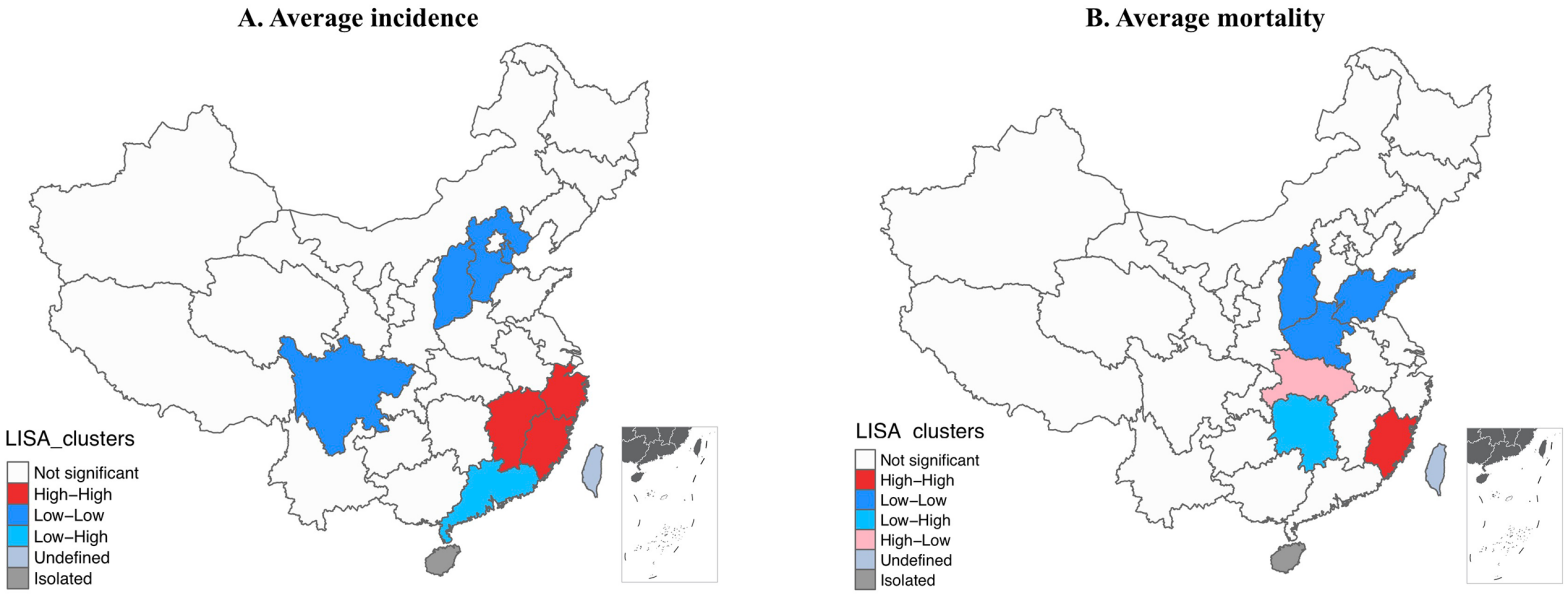
Local cluster analysis of hepatitis of unspecified aetiology incidence and mortality rates per 100,000: 2004–2020. **(A)** Incidence rate. **(B)** Mortality rate. Grayscale and color categories correspond to deciles of the histogram of displayed values. LISA, local indicators of spatial association.

Moreover, we explored the spatial autocorrelation of the incidence and mortality rates of HUA among the 31 provinces over these years. For the average annual incidence of HUA from 2004 to 2020, the global Moran’s *I* was 0.2082 (*p* < 0.001), which indicated that there was positive global spatial autocorrelation and that the incidence of HUA might not be randomly distributed in 31 provinces. Moreover, local Moran’s clusters of the incidence of HUA showed that Zhejiang, Fujian, Jiangxi, and their adjacent provinces had a higher average annual incidence in the high-high cluster. By contrast, Hebei, Tianjin, Shanxi, Sichuan, and their adjacent provinces had a lower average annual incidence in the low-low cluster (Figure 4A). The global Moran’s *I* of the average annual mortality rates of HUA was 0.05827 (*p* = 0.21), denoting that there was not a significant global spatial autocorrelation and that the mortality rates of HUA might be spread randomly throughout the country. Nevertheless, there was some local spatial autocorrelation according to the local Moran’s statistic (Figure 4B). Fujian and its adjacent provinces had a higher average annual mortality rate in the high-high cluster, while Henan, Shandong, Shanxi, and their adjacent provinces had a lower average annual mortality rate in the low-low cluster.

### Age distribution of HUA incidence and mortality, 2004–2020

**Figure 5:**
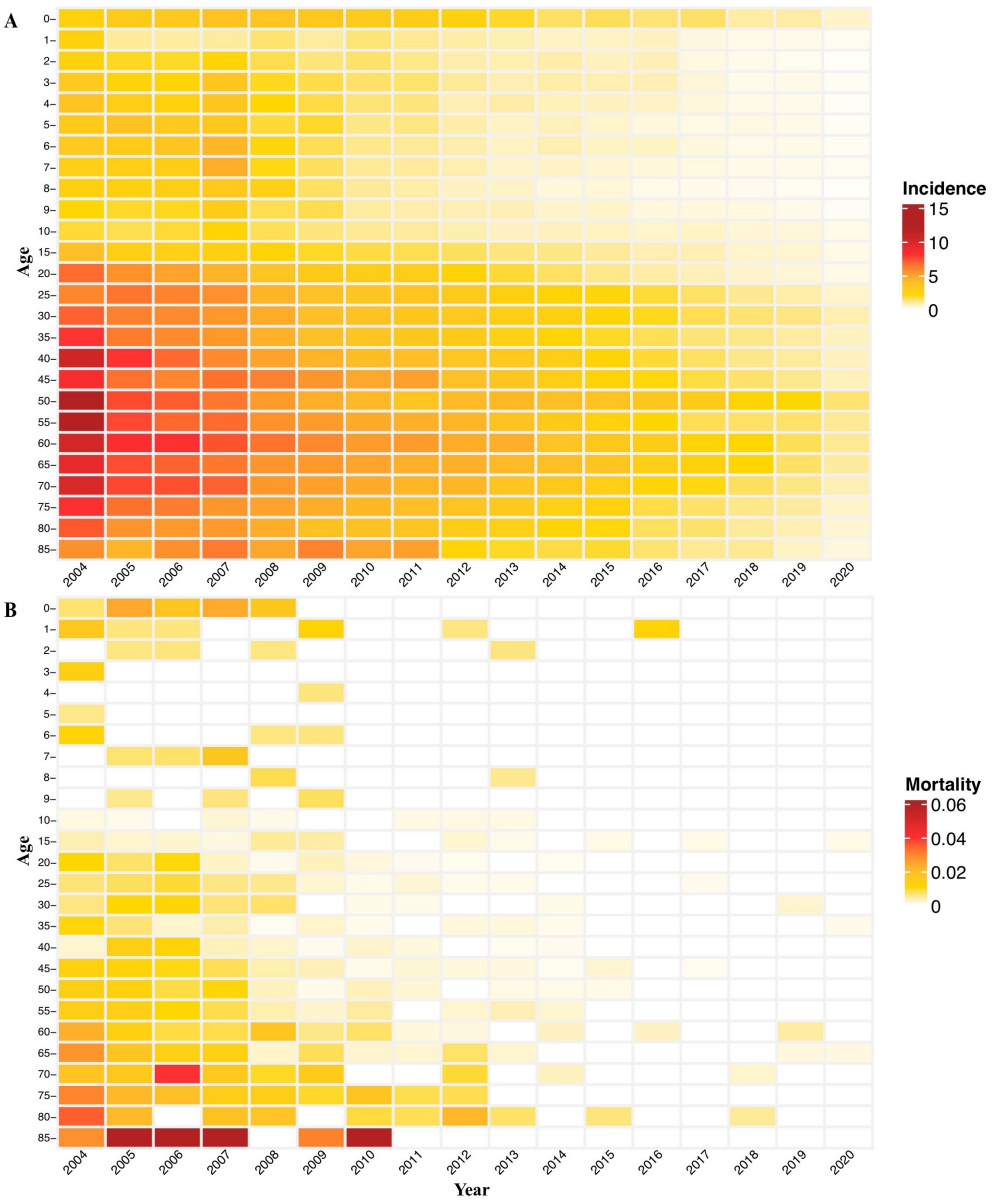
Age-specific yearly incidence and mortality of hepatitis of unspecified aetiology in China: 2004–2020. **(A)** Age-specific incidence by age group. **(B)** Age-specific mortality by age group.

**Figure 6:**
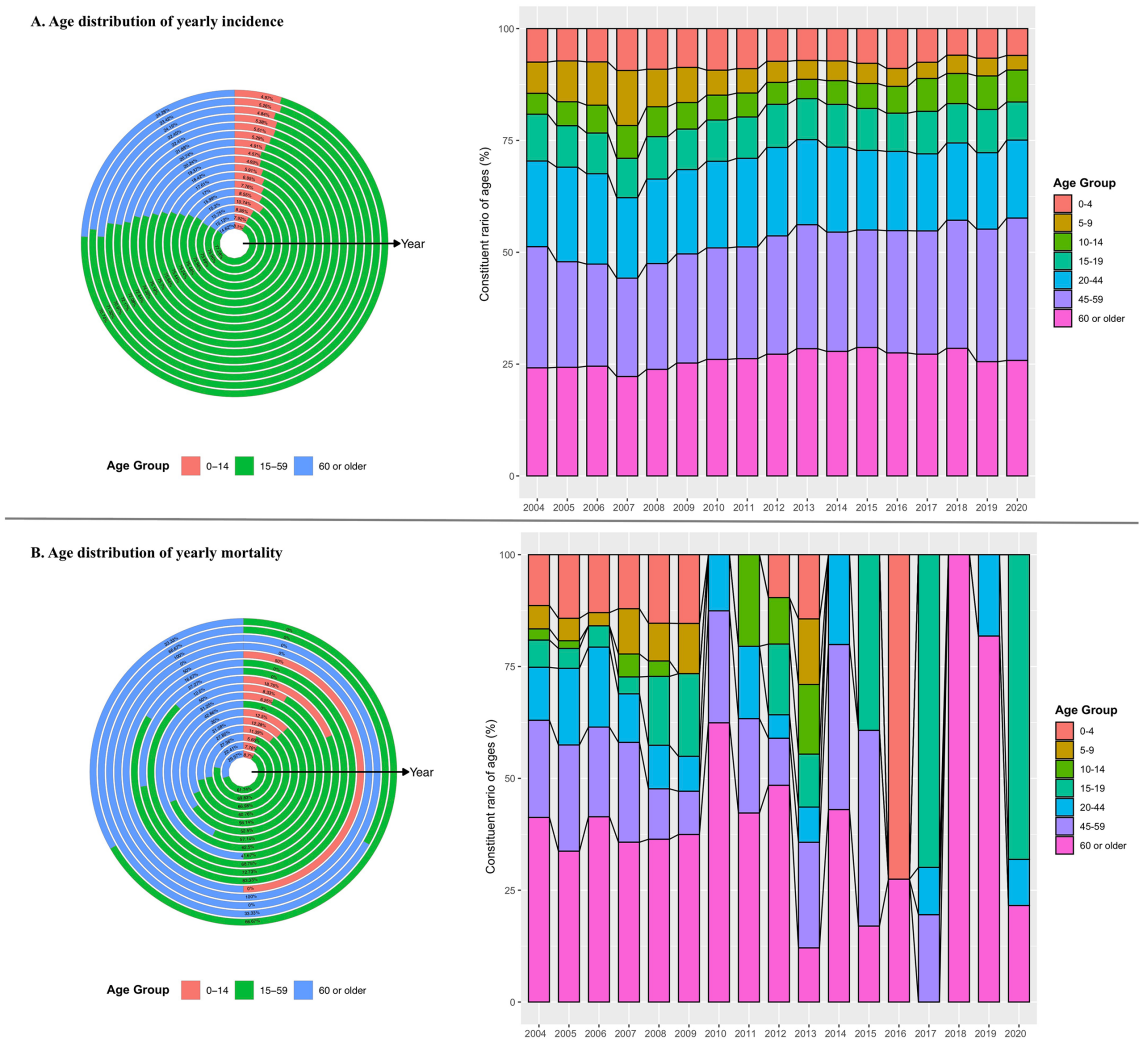
Age distribution of yearly incidence and mortality hepatitis of unspecified aetiology in China: 2004–2020. **(A)** Age-specific incidence and the incidence constituent ratio of ages. **(B)** Age-specific mortality and the mortality constituent ratio of ages.

From 2004 to 2020, the annual incidence among all age groups decreased steadily, falling to its lowest level in 2020 (Figure 5A). During this time, more than 70% of cases were seen in people aged 15–59 years old, followed by people aged 60 years or older (19%) and those 14 or younger (6%; Figure 6A). Moreover, there were no obvious incidence changes in the constituent ratio of ages, especially in teenaged groups (Figure 6A).

The annual mortality rate among people aged over 85 years was relatively higher from 2005 to 2007 and in 2010 (Figure 5B). In addition, over the past 17 years, the age-specific mortality rate due to HUA was highest in people aged 15–59 years (approximately 56%), followed by people 60 years or older (36%) and people 14 years or younger (8%; Figure 6B). It is noteworthy that the age group with the highest death constituent ratio was teenagers 15–19 years old in 2020, during the COVID-19 pandemic (Figure 6B).

### Correlations between HUA and other viral hepatitis, 2016–2020

**Figure 7:**
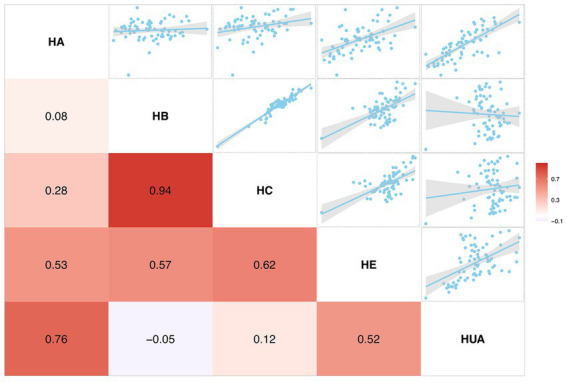
Correlations of monthly incidence rates between hepatitis of unspecified aetiology and other viral hepatitis: 2016–2020. *p* value indicates the coefficient of the Pearson correlation. HA, hepatitis A; HB, hepatitis B; HC, hepatitis C; HE, hepatitis E; and HUA, hepatitis of unspecified aetiology.

To better understand the epidemic characteristics of HUA, we further explored the associated correlation between HUA and other common types of viral hepatitis by using the available data on monthly incidence rates of the five hepatitis types from 2016 to 2020. The correlogram showed that the monthly incidence of HUA has a linear dependence relationship with the counterparts of hepatitis A and hepatitis E; the Pearson correlation coefficients were 0.76 (*p* < 0.001) and 0.52 (*p* < 0.001), respectively, which indicated that there were noteworthy positive correlations between them (Figure 7). We also constructed linear regression for modeling the relationship between the monthly incidence of HUA and hepatitis A (hepatitis E), with multiple R-squared of 0.58 (*p* < 0.001) and 0.27 (*p* < 0.001) separately. In contrast to hepatitis A and hepatitis E, the monthly incidence of hepatitis B (−0.05, *p* = 0.67) and hepatitis C (0.12, *p* = 0.31) were not relevant to HUA (Figure 7).

## Discussion

Major special national science and technology projects for the prevention and control of viral hepatitis in China were initiated in 2009. Our study describes the epidemiological features and trends of HUA from 2004 to 2021 (before and after 2009). We found that both the annual incidence of HUA and mortality rates due to HUA decreased sharply from 2004 to 2021 (all *p* < 0.001). These achievements are significant steps toward the goal of eliminating hepatitis in China by 2030. To our knowledge, our analysis covers the longest period and largest sample size studied on HUA in mainland China.

The incidence and mortality rates of HUA found in this study showed considerable downward trends from 2004 to 2021, possibly due to the following reasons. First, the World Health Organization (WHO) developed ambitious targets for the elimination of the hepatitis B virus (HBV) and hepatitis C virus (HCV) as public health threats by 2030. Over the past 2 decades, China, which has participated in this program, has made great progress in hepatitis prevention through increased national investment in vaccination and health education programs (27). All these efforts led to a decrease in cases of all types of viral hepatitis. For example, implementing vaccination programs for the general population was indeed an effective way to prevent viral hepatitis, although implementation was universal only for hepatitis A and B (2). Substantial vaccination programs not only played an essential role in easing the heavy burden of viral hepatitis but also yielded accessorial benefits for the decline of HUA (2, 28). Second, fecal-oral transmitted infectious diseases, such as hepatitis A and E, can spread through contaminated food and water or poor sanitary conditions. After undergoing rapid demographic and epidemiological changes in the past few decades, the Chinese government attached great importance to hygienic conditions, in response to the WHO’s appeal, by promoting the use of sanitary toilets and proper handwashing (29–31). Having experienced the ordeals of SARS (32), hand, foot and mouth disease (33), and the H1N1 pandemic (34), the public also gradually gained hygienic awareness. Third, with the advancement of medical technology, more state-of-the-art detection methods were presented and applied to diagnose cases of viral hepatitis A, B, C, D, and E (35, 36). Those patients with HUA would benefit greatly from the early diagnosis of specific hepatitis viruses on account of the increasing capabilities of virus detection techniques. Fourth, improved quarantine regulations and mechanisms might contribute to the significant decline in HUA cases (37). Fifth, the decrease in 2020 may be related to fewer people seeking healthcare and being tested for viral hepatitis during the COVID-19 pandemic (38).

For the spatial distribution, the annual incidence in all 31 provinces generally showed downward trends from 2004 to 2020. Fujian had the highest incidence of the 31 provinces almost every year from 2004 to 2020. Furthermore, Fujian and its adjacent provinces had a higher average annual incidence and belonged to the high-high cluster of spatial autocorrelation analysis. Epidemiological evidence showed that raw seafood was a dominant cause of enteric virus–related diseases (39, 40). For instance, the largest outbreak (over 300,000 cases were reported) of hepatitis A in Shanghai, China, in 1988, was caused by the consumption of clams grown and harvested from contaminated waters (41); similar incidents were also reported in Italy (42), Australia (43), Portugal (44), and other countries. Fujian was a coastal province located in southeast China, where seafood was a major dietary component for residents. Not surprisingly, these coastal settlements had a higher incidence of HUA because of their dietary habit. In contrast to central and western regions with lower incidence, the high incidence in coastal cities was a hint that government workers should pay more attention to the inspection and detection of seafood safety and water quality. From this event, we can also borrow the perspective that the diagnostic efficiency of viral hepatitis can be improved by auxiliary detection of viruses from food or water.

As to age distribution, people 15–59 years of age had more reported cases, accounting for nearly 70% of all HUA patients. However, the annual incidence decreased steadily irrespective of age group, falling to an all-time low level in recent years. Although the annual mortality rate of HUA always remained very low compared with the corresponding incidence, patients 85 years or older had a relatively higher annual mortality rate between 2005 and 2010. While there were no obvious incidence changes in the constituent ratio of ages in these years, the age group with the highest death constituent ratio was teenagers 15–19 years old in 2020, during the COVID-19 pandemic. This fact needs to be pursued by further studies.

After exploring temporal, spatial, and age-oriented attributes, we verified the similarity of HUA to other types of viral hepatitis. Some studies have reported that most unspecified hepatitis cases corresponded to hepatitis A cases in India and Panama (45, 46). As mentioned above, we also obtained an analogous result that a significantly large correlation exists between the monthly incidence of HUA and hepatitis A and hepatitis E, with Pearson correlation coefficients of 0.76 and 0.52, respectively, indicating strong relationships between them. Specifically, the monthly incidence of hepatitis A accounted for 58% of the variation in HUA, representing a distinct linear dependence on hepatitis A. One might reasonably consider whether most cases of HUA were derived from undiagnosed hepatitis A.

In this study of HUA in China from 2004 to 2021, we used national-level data covering 31 provinces and ages from 0 to over 85 years, comprehensively demonstrating the epidemiological characteristics of HUA in mainland China. However, our study still has several limitations. First, the data quality might vary because of changing case definitions over 18 years with dynamic diagnostic criteria. Second, we described the specific features of temporal, spatial, and age attributes of HUA but not other detailed demographic characteristics such as gender, occupation, nationality, and urban (rural) attributes. We will continue to carry out in-depth research in the future.

## Conclusion

Based on the 18-year national surveillance data, we found that overall HUA incidence and mortality decreased since 2004 and continued to do so in 2020–2021 during the COVID-19 pandemic (*p* < 0.001). All 31 provinces of mainland China saw a remarkable decline in HUA incidence and mortality. The proportion of HUA within viral hepatitis also trended downward trend from 2004 to 2021. However, no change was observed in the seasonal pattern or demographic features of cases during this decline. In particular, the incidence of HUA did not rise in the children’s group during the COVID-19 pandemic.

Tens of thousands of people are newly infected with HUA every year in China, and it is still a serious public health threat. Although the global epidemic of unexplained hepatitis in children is ongoing, we need to monitor trends of HUA in China, to improve HUA public health policy and practice. All these efforts will contribute toward achieving the goal of eliminating viral hepatitis by 2030.

## Data availability statement

The original contributions presented in the study are included in the article/Supplementary material, further inquiries can be directed to the corresponding authors.

## Ethics statement

Our analysis is approved by Zhejiang Provincial Center for Disease Control and Prevention. All the study was reviewed by the Research Ethics Committee and it is found that utilization of disease surveillance did not require oversight by an ethics committee.

## Author contributions

SL and SW conceived the study design. NZ and XG collected the data, developed statistical models, performed data analysis, and drafted the manuscript. LW, HZ, LG, ZM, YC, SQ, and ZY helped to collect the data. All authors contributed to the article and approved the submitted version.

## Funding

This research was supported by National Natural Science Foundation of China (31901120), China Postdoctoral Science Foundation (2022M723135), Beijing Natural Science Foundation (5192016), Emergency research project of novel coronavirus infection of Anhui province (2022e07020071), and Zhejiang Provincial Program for the Cultivation of High-Level Innovative Health Talents and Anhui Normal University Program for High-Level Talents.

## Conflict of interest

The authors declare that the research was conducted in the absence of any commercial or financial relationships that could be construed as a potential conflict of interest.

## Publisher’s note

All claims expressed in this article are solely those of the authors and do not necessarily represent those of their affiliated organizations, or those of the publisher, the editors and the reviewers. Any product that may be evaluated in this article, or claim that may be made by its manufacturer, is not guaranteed or endorsed by the publisher.

## Footnotes

^1^ http://www.nhc.gov.cn/cms-search/wsbz/wsbSearchList.htm

^2^ https://www.ncmi.cn/index.html

^3^ http://www.stats.gov.cn/index.html

^4^ http://www.satscan.org/

## Abbreviations

HUA: Hepatitis of unspecified aetiology
APC: Annual percentage change
CI: Confidence interval
