# Supplementary material for: Changing spatiotemporal patterns for hepatitis of unspecified aetiology in China, 2004–2021: a population-based surveillance study
Source: Front Public Health. 2023 May 5;11:1177965. doi: 10.3389/fpubh.2023.1177965 (PMC10196104; doi:10.3389/fpubh.2023.1177965)
Supplement: Supplementary file 1 [file Image_1.pdf]

Supplementary files

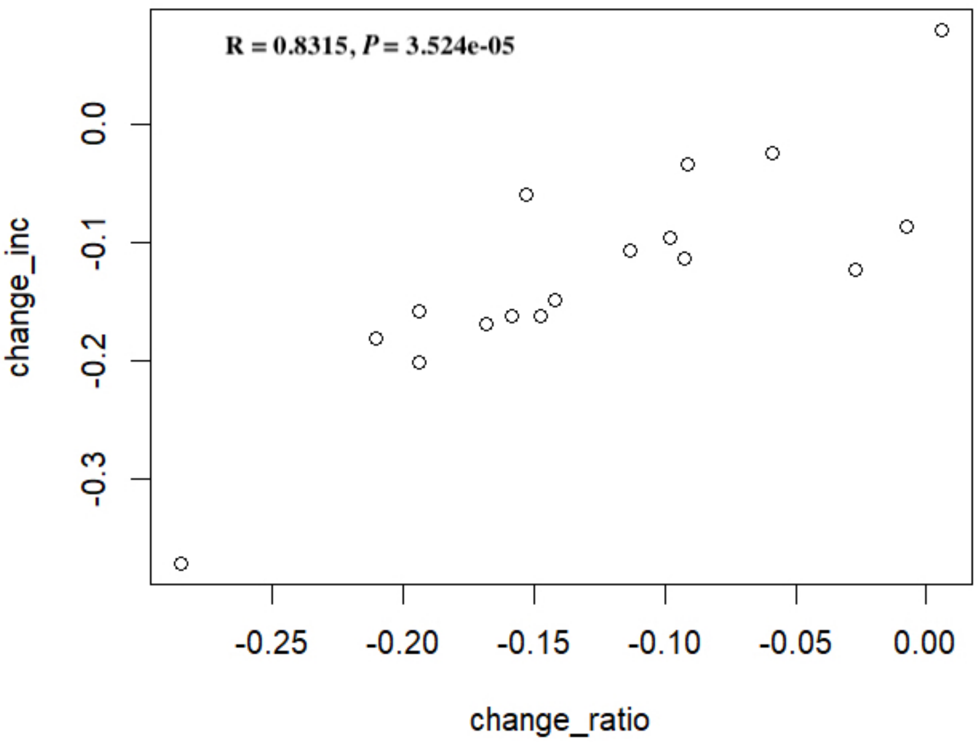

Supplementary figure 1. The correlation analysis between the change of HUA incidence rate and the change of constituent ratio of etiological confirmed cases, 2004-2021.
